# A Personalized, Transdiagnostic Smartphone Intervention (Mello) Targeting Repetitive Negative Thinking in Young People With Depression and Anxiety: Pilot Randomized Controlled Trial

**DOI:** 10.2196/47860

**Published:** 2023-12-13

**Authors:** Imogen Bell, Chelsea Arnold, Tamsyn Gilbertson, Simon D’Alfonso, Emily Castagnini, Nicola Chen, Jennifer Nicholas, Shaunagh O’Sullivan, Lee Valentine, Mario Alvarez-Jimenez

**Affiliations:** 1 Orygen Melbourne Australia; 2 Centre for Youth Mental Health University of Melbourne Melbourne Australia; 3 School of Computing and Information Systems University of Melbourne Melbourne Australia

**Keywords:** repetitive negative thinking, rumination, anxiety, depression, mobile app, just-in-time adaptive interventions, youth mental health, adolescent, mobile phone

## Abstract

**Background:**

Repetitive negative thinking (RNT) is a key transdiagnostic mechanism underpinning depression and anxiety. Using “just-in-time adaptive interventions” via smartphones may disrupt RNT in real time, providing targeted and personalized intervention.

**Objective:**

This pilot randomized controlled trial evaluates the feasibility, acceptability, and preliminary clinical outcomes and mechanisms of Mello—a fully automated, personalized, transdiagnostic, and mechanistic smartphone intervention targeting RNT in young people with depression and anxiety.

**Methods:**

Participants with heightened depression, anxiety, and RNT were recruited via social media and randomized to receive Mello or a nonactive control over a 6-week intervention period. Assessments were completed via Zoom sessions at baseline and at 3 and 6 weeks after baseline.

**Results:**

The findings supported feasibility and acceptability, with high rates of recruitment (N=55), uptake (55/64, 86% of eligible participants), and retention (52/55, 95% at 6 weeks). Engagement was high, with 90% (26/29) and 59% (17/29) of the participants in the Mello condition still using the app during the third and sixth weeks, respectively. Greater reductions in depression (Cohen *d*=0.50), anxiety (Cohen *d*=0.61), and RNT (Cohen *d*=0.87) were observed for Mello users versus controls. Mediation analyses suggested that changes in depression and anxiety were accounted for by changes in RNT.

**Conclusions:**

The results indicate that mechanistic, targeted, and real-time technology-based solutions may provide scalable and effective interventions that advance the treatment of youth mental ill health.

**Trial Registration:**

Australian New Zealand Clinical Trials Registry ACTRN12621001701819; http://tinyurl.com/4d3jfj9f

## Introduction

Approximately one-fifth of young people worldwide are affected by mental ill health [1,2], with anxiety and depression representing the most prevalent mental health conditions and the highest burden of disease [2-4]. With stagnating treatment effect sizes [5], poor engagement [6,7], and comorbidity rates as high as 76% among emotional disorders [8,9], the development of more targeted and effective treatments that meet young people’s needs and preferences is a top international research priority [10]. Accordingly, many have called for a paradigm shift toward a transdiagnostic approach to conceptualize and treat mental health disorders [11,12]. Transdiagnostic mechanisms are processes that underpin a range of mental health conditions, providing potentially more potent treatment targets compared with overarching syndromes that are broad, overlapping, and lacking in specificity [12-14].

Repetitive negative thinking (RNT) is a key transdiagnostic mechanism that accounts for the overlap between several mental disorders, particularly depression and anxiety [15,16]. RNT includes thoughts that are passive and hard to control and captures both worry (concern for future undesirable events) and rumination (excessive negative thoughts about the past or one’s depressive symptoms) [15]. In adolescent populations, heightened levels of RNT are strongly related to depression and anxiety [17] and predict the later onset of emotional disorders [18,19]. Furthermore, meta-analytic findings indicate that improvements in RNT following psychological treatment are related to improvements in depression and anxiety in both adults [20,21] and youth [22]. While reducing RNT may be associated with improvements in symptoms, current treatments may be improved using novel approaches that target RNT in everyday life.

A key shortcoming of existing psychological treatments is the inability to tailor interventions according to person, place, and time. Psychological problems and associated transdiagnostic processes are dynamic, showing a high degree of temporal variability as they unfold from moment to moment in interaction with the internal and external environments [23]. In contrast, treatments are typically delivered in brief and infrequent windows of time in a clinical setting, subsequently relying on the individual to remember when and how techniques should be used in daily life. Assessment methods also rely on the recollection of often subtle momentary experiences, potentially missing nuanced dynamics that could be relevant for tailoring interventions [24,25]. This “therapy–real world gap” [26] can result in a “one-size-fits-all” approach that fails to provide relevant support at the time, place, and frequency needed to effectively intervene and modify symptoms and underlying mechanisms as they arise.

RNT treatments typically involve skills and strategies aimed at disrupting the process as it is happening through techniques such as mindfulness, cognitive restructuring, or problem-solving [22,27]. However, these approaches suffer from the same limitations as other treatments in that they are most commonly taught in clinical settings, away from the contexts in which the problem actually occurs. Furthermore, although the evidence suggests that multiple strategies are likely to be effective for reducing RNT overall, there is a lack of knowledge on what works best for whom and in what context. For example, mindfulness may not suit some young people or it may be best suited to certain contexts. Hence, clinicians working in routine practice tend to adopt a flexible, formulation-driven approach that allows for tailoring intervention strategies to suit the individual presentation and adapt it based on needs over time [28,29]. Therefore, a personalized approach to RNT treatment that not only targets and delivers intervention at the moment RNT is happening but also tailors intervention strategies to individual characteristics and changing needs may offer a promising approach for improving outcomes.

Researchers have looked toward smartphone apps as a medium to deliver interventions in daily life, potentially overcoming the therapy–real world gap [30]. Research has found that smartphone-based interventions can be used to effectively treat a range of mental disorders in adults [31] and youth [32,33]. Young people are native adopters of technology [34] and demonstrate high levels of interest and motivation to use digital technologies, including apps, to manage their mental health [35-37].

Smartphone apps may improve the efficacy of transdiagnostic treatments through their unique capability to detect and respond to momentary changes in transdiagnostic mechanisms in real time using personalized interventions [38]. Just-in-time adaptive interventions (JITAIs) [39] harness mobile technologies such as smartphones to deliver tailored interventions at key moments using time-varying contextual data such as location or mood states. Contextual information is typically captured using ecological momentary assessment (EMA) [40], which involves repeated assessment of experiences in daily life, either passively through mobile sensors or actively via self-report. Critically, JITAIs allow adaptive tailoring of the intervention based on contextual variables to precisely time delivery to moments of need. Although JITAIs have only recently been defined and studied, meta-analyses have shown promising effects for mental health conditions [41]. Similar to JITAIs, but with less focus on tailoring to moments of need, ecological momentary interventions have also demonstrated positive effects for improving mental health and well-being outcomes [42].

The opportunity to target transdiagnostic mechanisms in real time using JITAIs is an area with significant potential to improve the personalization, engagement, and effectiveness of psychological interventions. The vast majority of JITAIs have focused on health behaviors, with very little research conducted on targeting key transdiagnostic mechanisms or for application in youth populations [41]. Findings from broader systematic reviews of smartphone interventions for youth have also failed to identify any interventions targeting transdiagnostic processes specifically [33]. As a result, many smartphone interventions suffer the same “one-size-fits-all” approach of traditional psychological treatments that cover a wide range of symptoms within broad disorder categories. Alternatively, by specifically targeting transdiagnostic mechanisms across a range of conditions, smartphone interventions may offer more relevance to a broader number of young people while also offering more personalized, engaging, and accessible support in daily life that is also highly scalable. Furthermore, modifying the transdiagnostic mechanisms underlying mental health symptoms may provide a more personalized and targeted intervention approach.

To the best of our knowledge, the potential for JITAIs to provide personalized interventions targeting disruption in transdiagnostic mechanisms in real time and real-world contexts for youth mental health has not yet been explored. To investigate this potential, our group developed a world-first smartphone intervention called “Mello,” a self-guided, personalized, transdiagnostic, and mechanistic smartphone intervention targeting RNT in young people with depression and anxiety. Mello is based on the JITAI model in that intervention strategies designed to disrupt RNT in the moment are tailored based on momentary assessments of relevant contextual variables. Mello does not adopt a single therapeutic modality but rather translates multiple techniques from third- and second-wave cognitive behavior therapies, which have evidence for reducing RNT into brief interventions designed to disrupt RNT in the moment. This was based on the rationale that effective intervention strategies exist across different therapeutic modalities, and consultations with young people and clinicians through the development of Mello clearly highlighted the range of techniques that were used to effectively reduce RNT in the moment. Consistent with the JITAI model of identifying and intervening in moments of need, Mello uses an algorithm that tailors a recommendation for a microintervention designed to disrupt RNT based on responses to a brief EMA check-in that captures the levels of RNT, mood, context, and location.

This study was a pilot randomized controlled trial (RCT) comparing Mello with a nonactive control group over a 6-week period. The primary aim was to evaluate the feasibility, acceptability, and estimated efficacy of Mello in reducing RNT, depression, and anxiety in youth with clinical levels of depression and anxiety and increased RNT. Secondarily, the exploratory aims were to evaluate whether the effect of Mello on depression and anxiety outcomes was explained by effects on RNT, as this was the transdiagnostic target of the intervention. Further aims were to evaluate engagement with Mello and the effect of the intervention on the secondary outcomes.

## Methods

### Study Design

This study was an open label, parallel group RCT. Participants were allocated on a 1:1 ratio to the Mello app intervention group or a nonactive control group.

### Participants

Participants were recruited through paid web-based social media advertisements on Facebook and Instagram. Participants were young people from the general population aged between 16 and 25 years who were experiencing clinical levels of depression and anxiety (≥10 on the Patient Health Questionnaire 8 [PHQ-8] [43] and Generalized Anxiety Disorder Scale 7 [GAD-7] [44]) and elevated RNT levels (≥37 on the Perseverative Thinking Questionnaire [PTQ] [45]). Cutoffs for the PHQ-8 and GAD-7 were chosen based on well-established clinical severity thresholds for indicating probable depressive disorder or generalized anxiety disorder [43,44]. Although the PTQ does not have published clinical norm data, a score of ≥37 was chosen based on the mean severity levels in clinical samples from prior research [45]. Participants also needed to be able to provide informed consent, have sufficient command of the English language, and own and use a smartphone capable of running the app (Android or iPhone). Participants were excluded if they were a current psychiatric inpatient or if they were receiving care from a crisis management team. There were no restrictions on access to other psychological or mental health treatments during trial participation.

A target sample size of 30 participants was initially deemed sufficient to meet the aims as a pilot study and is consistent with the guidelines in the literature for pilot trials [46-48] and similar pilot RCTs of smartphone interventions [49,50].

### Randomization and Blinding

Participants were randomized to the Mello or a control group using a computer-generated randomization table, with block sizes of 6 and 8, and stratified based on whether the participant was receiving psychological or other mental health treatment at the time of study participation. The randomization table was created by a researcher independent of the research team and uploaded into REDCap (Research Electronic Data Capture; Vanderbilt University). Researchers randomized participants following the completion of informed consent and baseline questionnaires using the REDCap application. Therefore, allocation was concealed from the researchers’ enrolling participants until the end of the baseline assessment. Owing to the nature of the intervention, the study participants were not blinded to the condition. As all outcomes were assessed via self-reported questionnaires, the researchers were also not blinded to the condition.

### Procedure

Study advertisements on social media linked to a web-based information and expression of interest page. Participants who submitted an expression of interest were sent information about the study (Multimedia Appendix 1) via email and contacted via phone by researchers to discuss the study and screen them for eligibility. Eligible and interested participants completed written and informed consent and self-report baseline questionnaires in a web-based Zoom session with a member of the research team. Participants were randomized to the condition following the completion of consent and baseline questionnaires. Those allocated to the Mello group downloaded the app on their own smartphones and were provided brief instructions on how to use the app. Participants in the Mello group received access to the Mello app for 6 weeks as well as weekly telephone calls from members of the research team. The phone calls were brief in nature (approximately ≤5 min in all cases) and involved general encouragement to use the app and troubleshooting of any technical issues. No therapeutic support was provided in these phone calls as the focus was on troubleshooting technical or usability issues. Participants were encouraged to complete the check-ins daily and therapy activities where relevant; however, they were told they could use the app as they chose during the 6-week intervention period. Participants in the nonactive control condition did not receive access to the Mello app or adjunct phone calls. At 3 and 6 weeks after baseline, all participants completed questionnaires via Zoom sessions with a member of the research team. Participants in the Mello group uninstalled the app from their phones in the final (6 weeks) assessment session.

### Interventions

#### Intervention Development

Mello was developed based on user-centered design principles [51] by a multidisciplinary team consisting of a clinician researcher, technology developers, and design experts, in consultation with groups of young people with lived experience of depression and anxiety and clinicians. The first phase involved a series of individual interviews and group workshops to understand the needs and experiences of RNT among young people with depression and anxiety as well as parallel sessions with clinicians to understand the experiences and approaches to reducing RNT in routine treatment. Concurrently, a literature review and meta-analysis were conducted to identify the existing treatment approaches for reducing RNT [22]. This work led to a detailed understanding of the experience of RNT, strategies that can be used to reduce RNT, and common barriers to treatment, all of which validated the concept and value proposition of Mello. In the next phase, the design and development team ran several further workshops with young people. The team consisted of a user experience designer, brand and user interface designer, and mobile app developer led by a researcher clinician (IB) and guided by an industry design agency and project funders. These sessions focused on mapping the architecture of the intervention and developing prototypes through wireframes. Through reviews of the clinical content and focus group sessions involving wireframes, the prototype was iteratively refined based on ongoing feedback. Simultaneously, the brand and user interface designer developed the brand identity and graphic design, which was validated and refined through feedback from young people. Finally, the app was built and iteratively tested with users to fix bugs and refine its usability.

#### Intervention Description

**Figure 1:**
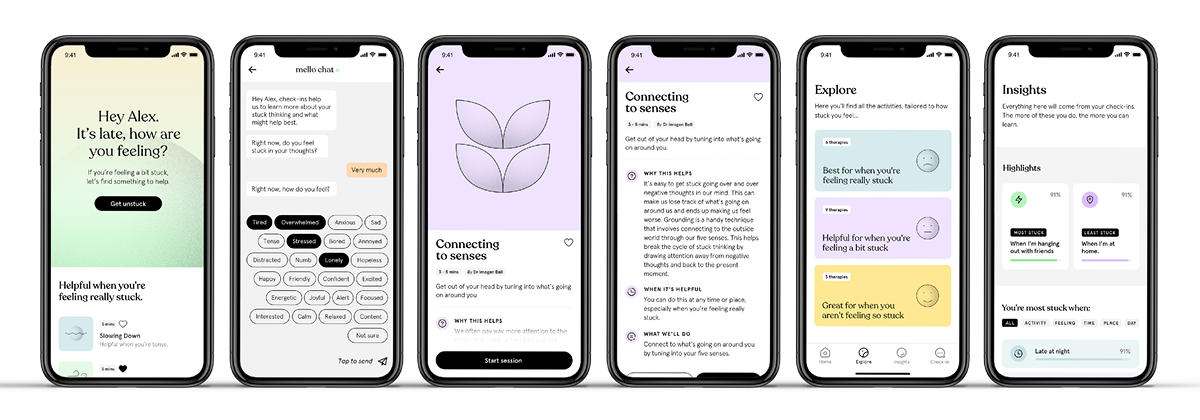
Mello app features.

The Mello app uses real-time assessments to tailor momentary intervention strategies targeting the disruption of RNT in real time for young people with depression and anxiety (Figure 1). Users received randomized notifications 3 times a day (between 9 AM and 9 PM) to complete a brief 4-item questionnaire (“check-ins”) via a chat interface capturing levels of RNT, mood, activities, and location. On the basis of responses to the check-in questions, the app recommends 1 of 12 cognitive behavioral therapy activities to complete in the moment (Multimedia Appendix 2). The recommendations are based on a preprogrammed algorithm that matches strategies to the level of stuck thinking (high or low), mood (positive or negative), context (at home or away from home), and activity (active or passive). Each therapy activity takes approximately 2 to 12 minutes and involves animations overlaid with guided text or audio in which the user is guided through an evidence-based strategy for reducing RNT. Activities were selected based on a recent meta-analysis [22] on psychological treatment approaches for reducing RNT and included exercises such as mindfulness, problem-solving, defusion, worry time, thought challenging, and self-compassion. After completing an activity, the user is guided back to the chat interface, where they are again asked about their level of RNT and how helpful the activity was. To provide insight into patterns of RNT, information collected via check-ins feeds into a profile section of the app, which provides insights into the association between RNT and contextual factors (mood, activity, and location) for each participant. In addition to access via check-ins, participants can access all therapy activities on demand and save their favorite activities.

### Outcomes

The primary outcomes were feasibility, acceptability, and preliminary efficacy of the intervention. Feasibility was assessed through trial uptake, attrition, and engagement. A preregistered target sample of 30 participants was set to meet the aims of the study, with feasibility indicated if this sample size was met within the 6-month recruitment timeframe. Acceptability was assessed with the subscales (range 1-5, with higher scores indicating more positive evaluations) of the Mobile Application Rating Scale–User version [52]. The primary mental health outcomes were depression, as measured by the PHQ-8 total score (range 0-24, with higher scores indicating more severe symptoms) [43]; anxiety, as measured by the GAD-7 total score (range 0-21, with higher scores indicating more severe symptoms) [44]; and RNT, as measured by the total score on the PTQ (range 0-60, with higher scores indicating more severe RNT) [45].

Secondary outcomes were rumination, as measured by the Ruminative Response Scale (RRS) total score (range 22-88, with higher scores indicating greater severity) [53]; worry, as measured by the Penn State Worry Questionnaire total score (range 16-80, with greater scores indicating more severe worry) [54]; cognitive defusion, as measured by the Cognitive Fusion Questionnaire (CFQ) total score (range 7-49, with higher scores indicating greater tendency to fuse with negative thoughts) [55]; cognitive reappraisal, as measured by the Emotion Regulation Questionnaire total score on the reappraisal subscale (range 6-42, with higher scores indicating greater use of cognitive reappraisal strategies to reduce negative thoughts or increase positive thoughts) [56]; and well-being, as measured by the total score on the Warwick-Edinburgh Mental Well-being Scale (WEMWBS) total score (range 14-70, with higher scores indicating greater well-being) [57]. Three single items measured self-awareness of patterns in RNT (“I am aware of patterns in my negative thinking”; “I am aware of the things that affect my negative thinking patterns”) and confidence in self-management of RNT (“I am confident in my ability to manage my negative thinking patterns”), using visual analog scales ranging from 0 (strongly disagree) to 100 (strongly agree). Finally, the digital therapeutic alliance was explored using the Digital Working Alliance Inventory total score (range 6-42 with higher scores in this study indicating a better working alliance) [58].

### Statistical Analysis

All data analyses were performed on an intention-to-treat basis. Generalized linear mixed models with a restricted maximum likelihood estimator implemented by the lme4 (version 1.1-26) package in R (version 4.0.4; R Foundation for Statistical Computing) were used to assess the associations between the condition and changes in clinical outcome variables over time (baseline, during the intervention, and after the intervention). The models included random intercepts for each participant; a random slope for time; and fixed effects of the treatment group, time, and treatment group-by-time interactions. Generalized linear mixed models were also used to examine whether changes in the mechanism measures of RNT may be associated with changes in depression and anxiety. Pearson correlations between engagement indices and clinical measures were calculated to explore the associations between app engagement and outcomes.

### Ethical Considerations

The study was approved by the University of Melbourne Human Research Ethics Committee (project 2021-22524-23553-5) and prospectively registered (ACTRN12621001701819). Reporting followed CONSORT (Consolidated Standards of Reporting Trials) guidelines (Multimedia Appendix 3). All the data were deidentified.

Participants provided written informed consent via the REDCap web application. All participants received a full explanation of the study, in lay terms, relating to the aims of the study, study procedures, and potential risks and benefits in taking part before providing informed consent. The participants were reminded that they can withdraw from the study at any time without prejudice. Participants were provided with a copy of the participant information and consent form and were reimbursed Aus $45 (US $29) for completing each assessment session. The participants were not reimbursed for their use of the Mello app.

## Results

### Feasibility

**Figure 2:**
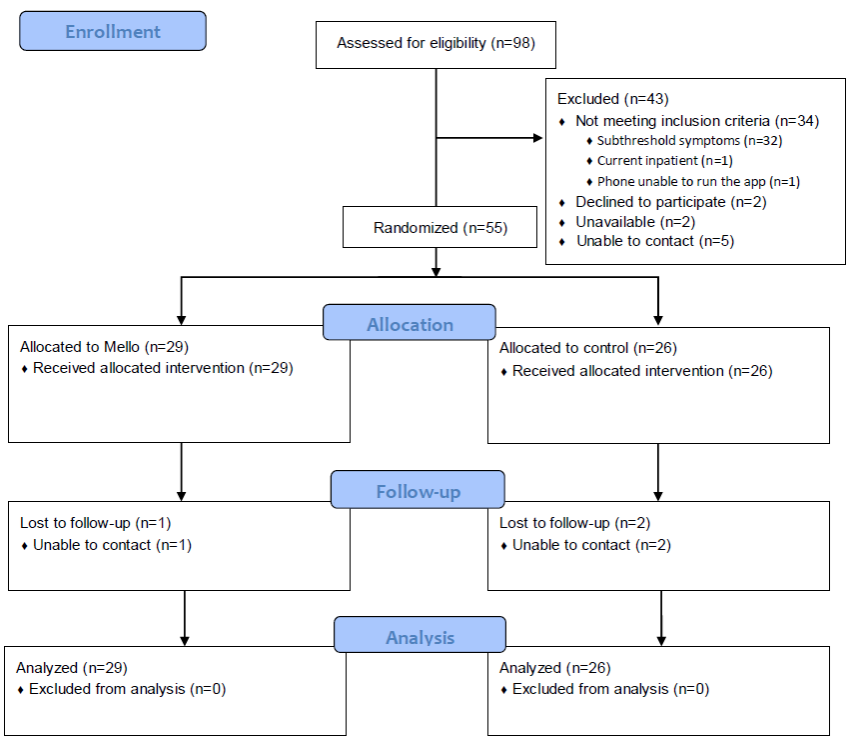
CONSORT (Consolidated Standards of Reporting Trials) flow diagram.

Participants were recruited from December 13, 2021, to March 8, 2022. The target sample size of 30 participants was recruited in ≤2 months, substantially faster than anticipated. As such, recruitment continued for the prespecified period of 6 months to enable more data to be captured, resulting in a final sample of 55 participants. As shown in Figure 2, a total of 98 participants were screened for eligibility. Of the 64 participants assessed as eligible, 55 (86%) consented and were randomized to the condition. Six-week follow-up data were available for 28 (97%) of the 29 participants in the Mello condition and 24 (92%) of the 26 participants in the control condition. For Mello users, 82% (143/174) of weekly phone calls were completed.

There were no deviations from the protocol and no updates to the app throughout the trial. Technical issues included 9 participants experiencing disruption to the log-in process, resulting in the participants having to create additional accounts to access the app.

Table 1 shows the baseline demographic characteristics of the participants. There were no significant differences between the groups in any of the baseline demographic or clinical characteristic variables (*P*>.05).

**Table 1:** Baseline participant characteristics.

Demographic variable			Control group (n=26)		Mello group (n=29)		Total (N=55)
**Gender, n (%)**							
	Men	5 (19)		5 (17)		10 (18)	
	Women	14 (54)		20 (69)		34 (62)	
	Gender diverse	7 (27)		4 (14)		11 (20)	
Age (y), mean (SD)			20.2 (2.6)		20.8 (2.8)		20.6 (2.7)
**Place of birth, n (%)**							
	Australia	19 (73)		21 (72)		40 (73)	
	Asia	5 (19)		6 (21)		11 (20)	
	New Zealand	1 (4)		0 (0)		1 (2)	
	Europe	1 (4)		0 (0)		1 (2)	
	North America	0 (0)		2 (7)		2 (4)	
Aboriginal or Torres Strait Islander, n (%)			1 (4)		1 (3)		2 (4)
**Highest educational qualification, n (%)**							
	Year 10 or less	2 (8)		2 (7)		4 (7)	
	Year 11 or12	17 (65)		20 (69)		37 (67)	
	Diploma	1 (4)		1 (3)		2 (4)	
	Bachelor’s degree	6 (23)		4 (14)		10 (18)	
	Master’s degree	0 (0)		2 (7)		2 (4)	
**Current occupation, n (%)**							
	Student	15 (58)		16 (55)		31 (56)	
	Casual or part-time employment	6 (23)		7 (24)		13 (24)	
	Full-time employment	0 (0)		1 (3)		1 (2)	
	Volunteer	0 (0)		1 (3)		1 (2)	
	Unemployed	5 (19)		4 (14)		9 (16)	
**Mental health service history, n (%)**							
	No history of service use	2 (8)		2 (7)		4 (7)	
	Previous service user	5 (19)		7 (24)		12 (22)	
	Current service user	19 (73)		20 (69)		39 (71)	
**Smartphone use, n (%)**							
	Uses several times/h	14 (54)		17 (59)		31 (56)	
	Uses every hour	10 (39)		7 (24)		17 (31)	
	Uses once—several times/d	2 (8)		5 (17)		7 (13)	
	Previously used smartphone apps for mental health	17 (65)		17 (59)		34 (62)	

### Acceptability

The mean Mobile Application Rating Scale–User version scores are listed in Table 2. All subscale scores were above 3.5 out of a possible 5, indicating generally positive evaluations. In addition, 96% (27/28) of the participants who used Mello reported that they would recommend the app to others. With regard to ongoing use, 43% (12/28) of the participants who used Mello reported that they would use the app 10 to 50 times in the next 12 months, and 36% (10/28) reported that they would use the app more than 50 times. Finally, 96% (27/28) of app users rated the Mello app as average (3 out of 5) or above, with 71% (20/28) rating it as 4 or 5 stars (out of 5; see Multimedia Appendix 4 for ratings of individual therapy activities). The average total score on the Digital Working Alliance Inventory was 29.5 (SD 6.5; see Multimedia Appendix 5 for scores on the individual items).

**Table 2:** User version of the Mobile App Rating Scale (uMARS; score range 1-5).

uMARS subscale (n=28)	Values, mean (SD)
Engagement	3.59 (0.53)
Functionality	4.18 (0.68)
Esthetics	4.37 (0.51)
Information	4.44 (0.47)
App quality	4.14 (0.43)
Perceived impact	3.77 (0.79)

### Engagement

All participants randomized to the Mello group downloaded the app and used it at least once. Over the 6-week intervention period, the 29 participants using Mello completed a total of 868 check-ins, with a mean of 29.93 (SD 18.06) out of a possible 126 check-ins. Participants completed 497 therapy activities, with a mean of 17.14 (SD 15.15) across the intervention period. At 3 weeks after baseline, 90% (26/29) of the participants were using the app and 59% (17/29) of the participants were still using the app during the sixth week of the intervention period (Table 3). On average, participants used Mello on nearly half (20.66/42, 49%) of the days during the 6-week intervention period (Table 3). Exploratory analyses did not reveal any significant associations between changes in PTQ, PHQ-8, or GAD-7 scores and indices of engagement (all *P*>.05; Multimedia Appendix 6).

**Table 3:** Engagement with the Mello app.

Engagement index		Mello users (n=29)
Number of therapy activities launched, mean (SD; range)		17.14 (15.15; 3-59)
Number of check-ins completed, mean (SD; range)		29.93 (18.06; 7-62)
Distinct days using Mello^a^, mean (SD; range)		20.66 (10.54; 6-38)
Percentage of days using Mello^b^, mean (SD; range)		49.18 (25.09; 14.3-90.5)
**Percentage users active per week, n (%)**		
	Week 1	29 (100)
	Week 2	27 (93)
	Week 3	26 (90)
	Week 4	22 (76)
	Week 5	19 (66)
	Week 6	17 (59)

^a^Total distinct days with some use of the Mello app over the 6-week intervention period.

^b^Percentage of days with some use of the Mello app over the 6-week intervention period.

### Clinical Outcomes

**Figure 3:**
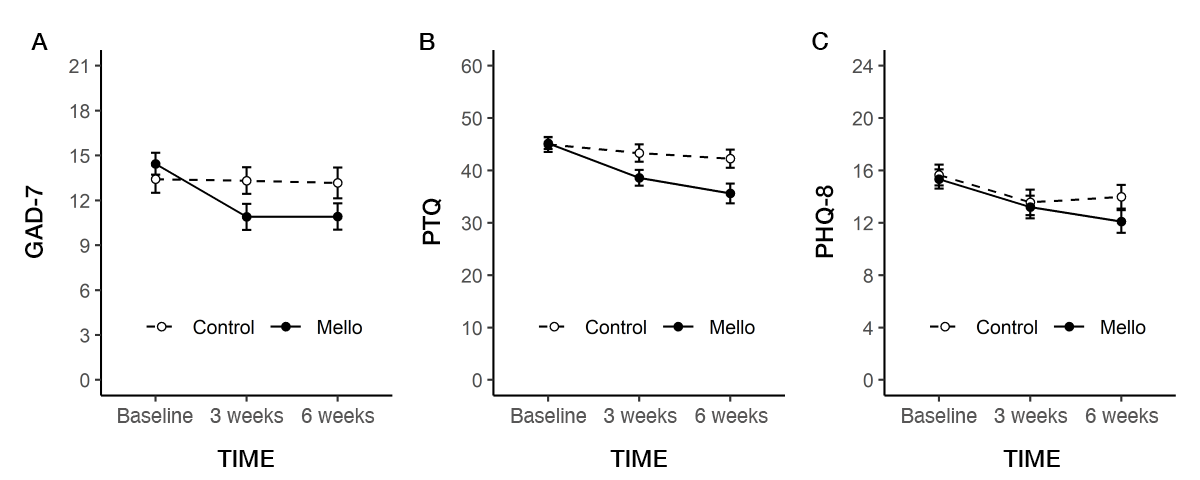
Primary outcomes at each time point per condition. (A) GAD-7: Generalized Anxiety Disorder 7; (B) PHQ-8: Patient Health Questionnaire 8; (C) PTQ: Perseverative Thinking Questionnaire.

Table 4 summarizes the mean scores, effect sizes, and group comparison analyses of the primary and secondary outcomes. There were significant group-by-time interaction effects for the primary outcomes of anxiety and RNT. Compared with the control group (*F*_1,23_=0.0015; *P*=.97), the Mello app users demonstrated a significant decrease in anxiety (GAD-7 total) from 0 to 6 weeks (*F*_1,27_=16.94; *P*<.001; Figure 3A). There was also a significant decrease in scores for the Mello group from 0 to 6 weeks (*F*_1,27_=28.25; *P*<.001), compared with the control group (*F*_1,23_=2.34; *P*=.14) for RNT (PTQ), with the Mello group displaying significantly lower scores than the control group at both 3 weeks (*F*_1,52_=4.42; *P*=.04) and 6 weeks (*F*_1,50_=6.59; *P*=.01; Figure 3B). A trend toward significant group-by-time interaction effects was observed for the final primary outcome, depression (PHQ-8; *F*_1,51_=3.21; *P*=.08); however, this was not statistically significant. However, there was a significant main effect of time, whereby scores decreased between baseline and 6 weeks for both the Mello and control groups (*F*_1,51_=23.74; *P*<.001; Figure 3C).

**Table 4:** Change in outcome variables between baseline to 6 weeks.

Outcome variable and time point				Control (n=26), mean (SE)		Mello (n=29), mean (SE)		*F* test (group×time interaction; *df*)		Cohen *d*		*P* value
**PHQ-8^a^**												
	Baseline			15.65 (0.79)		15.34 (0.73)		N/A^b^		N/A		N/A
	3 weeks			13.56 (0.97)		13.21 (0.87)		N/A		N/A		N/A
	6 weeks			14 (0.9)		12.11 (0.86)		3.21 (1,51)		0.50		.08
**GAD-7^c^**												
	Baseline			13.42 (0.91)		14.45 (0.73)		N/A		N/A		N/A
	3 weeks			13.32 (0.89)		10.9 (0.88)		N/A		N/A		N/A
	6 weeks			13.17 (1.03)		10.93 (0.88)		9.06 (1,51)		0.61		.004
**PTQ^d^**												
	Baseline			44.96 (1.41)		45.21 (1.12)		N/A		N/A		N/A
	3 weeks			43.32 (1.65)		38.62 (1.51)		N/A		N/A		N/A
	6 weeks			42.25 (1.74)		35.61 (1.88)		9.59 (1,51)		0.87		.003
**RRS^e^**												
	Baseline			65.38 (2.19)		66.31 (1.8)		N/A		N/A		N/A
	3 weeks			63.84 (2.26)		58.45 (1.91)		N/A		N/A		N/A
	6 weeks			61.17 (2.44)		56.25 (2.54)		4.35 (1,51)		0.59		.04
**PSWQ^f^**												
	Baseline			69.23 (1.66)		67.69 (1.27)		N/A		N/A		N/A
	3 weeks			67.48 (1.61)		63.17 (1.39)		N/A		N/A		N/A
	6 weeks			67.62 (1.38)		61.18 (1.93)		3.74 (1,51)		0.54		.06
**CFQ^g^**												
	Baseline			41.04 (0.98)		40.41 (0.85)		N/A		N/A		N/A
	3 weeks			40.56 (1.02)		36.45 (0.95)		N/A		N/A		N/A
	6 weeks			38.92 (1.12)		33.79 (1.57)		5.85 (1,51)		0.67		.02
**ERQ reappraisal^h^**												
	Baseline			21.88 (1.9)		21.1 (1.41)		N/A		N/A		N/A
	3 weeks			23.48 (1.52)		22.07 (1.34)		N/A		N/A		N/A
	6 weeks			21.67 (1.73)		23.32 (1.41)		1.66 (1,51)		0.36		.20
**WEMWBS^i^**												
	Baseline			37.35 (1.49)		36.59 (1.07)		N/A		N/A		N/A
	3 weeks			38.8 (1.86)		38.07 (1.07)		N/A		N/A		N/A
	6 weeks			37.12 (1.92)		41.32 (1.33)		4.60 (1,51)		0.61		.04
**SIS^j^**												
	**Thoughts**											
		Baseline	74.77 (2.85)		76.9 (4.35)		N/A		N/A		N/A	
		3 weeks	74.84 (3.37)		75.9 (3.31)		N/A		N/A		N/A	
		6 weeks	74.21 (3.95)		77.18 (3.19)		0.13 (1,51)		0.10		.73	
	**Factors**											
		Baseline	67.62 (3.9)		69.28 (4.41)		N/A		N/A		N/A	
		3 weeks	67.04 (5.69)		74.79 (3.29)		N/A		N/A		N/A	
		6 weeks	69.38 (4.74)		75.07 (2.62)		0.47 (1,51)		0.19		.49	
	**Confidence**											
		Baseline	28.96 (3.78)		30.55 (4.1)		N/A		N/A		N/A	
		3 weeks	34.8 (4.54)		42.79 (4.12)		N/A		N/A		N/A	
		6 weeks	28.92 (3.88)		49.04 (4.04)		17.27 (1,51)		1.66		<.001	

^a^PHQ-8: Patient Health Questionnaire 8.

^b^N/A: not applicable.

^c^GAD-7: Generalized Anxiety Disorder 7.

^d^PTQ: Perseverative Thinking Questionnaire.

^e^RRS: Ruminative Response Scale.

^f^PSWQ: Penn State Worry Questionnaire.

^g^CFQ: Cognitive Fusion Questionnaire.

^h^ERQ reappraisal: Emotion Regulation Questionnaire Reappraisal subscale.

^i^WEMWBS: Warwick-Edinburgh Mental Well-being Scale.

^j^SIS: single item self-awareness and self-management measures.

There were significant group-by-time interaction effects for the secondary outcome variables of rumination (RRS total), cognitive defusion (CFQ total), well-being (WEMWBS total), and confidence in self-management of RNT (single item self-awareness and self-management measures: confidence; Multimedia Appendix 7). Post hoc tests revealed that there was a significant decrease in RRS total scores for the Mello group from 0 to 6 weeks (*F*_1,27_=14.69; *P*<.001), compared with the control group (*F*_1,23_=3.31; *P*=.08). CFQ total scores were significantly lower in the Mello group than in the control group at 3 weeks (*F*_1,52_=8.67; *P*=.005) and 6 weeks (*F*_1,50_=6.65; *P*=.01). There were also significant decreases in CFQ total scores for both the Mello group (*F*_1,27_=16.91; *P*<.001) and the control group (*F*_1,23_=7.39; *P*=.01) over time. There was a significant increase in WEMWBS total scores in the Mello group between 0 and 6 weeks (*F*_1,27_=8.49; *P*=.007), compared with the control group (*F*_1,23_=0.14; *P*=.71). For the single item measuring confidence in self-management of RNT, there was a significant increase in scores for the Mello group between 0 and 6 weeks (*F*_1,27_=31.92; *P*<.001) compared with the control group (*F*_1,23_=0.03; *P*=.86), with the Mello group displaying significantly higher scores than the control group at 6 weeks (*F*_1,50_=12.66; *P*<.001). Changes in other secondary outcome variables did not differ between the groups from baseline to 6 weeks (Table 4; Multimedia Appendix 7). However, a main effect of time was found for worry (Penn State Worry Questionnaire total; *F*_1,51_=11.86; *P*=.001), where scores for both groups decreased between baseline and 6 weeks. No main effects were observed for this group.

### Mechanisms

Exploratory analyses were conducted to explore whether changes in the mechanism of RNT may be associated with changes in the primary outcomes of depression and anxiety. As previously stated, a significant interaction effect was observed between groups over time for anxiety (*F*_1,51_=9.06; *P*=.004) and RNT (*F*_1,51_=9.59; *P*=.003; Table 4). When RNT was added to the model with anxiety as an outcome, there was evidence of a mediation effect, as RNT had an effect on anxiety (*F*_1,137_=33.09; *P*<.001), but the group-by-time interaction effect was no longer significant (*F*_1,55_=0.08; *P*=.78).

In terms of depression, a trend toward significant group-by-time interaction was observed (*F*_1,51_=3.21; *P*=.08; Table 2). When RNT was added to the model with depression as an outcome, there was evidence of a mediation effect, as RNT had an effect on depression (*F*_1,136_=73.06; *P*≤.001) and the group-by-time interaction effect was clearly nonsignificant (*F*_1,55_=1.26; *P*=.27). These exploratory results suggest that changes in depression and anxiety may be explained by RNT.

### Adverse Events

Two participants in the Mello group reported mild adverse events: one with worsening mental health and the other with physical pain, both deemed unrelated to the intervention or study participation. No serious or trial-related adverse events were reported during the trial.

## Discussion

### Principal Findings

The aim of this pilot RCT was to evaluate the feasibility, acceptability, and estimated efficacy of a personalized, transdiagnostic, and mechanistic smartphone intervention called Mello for targeting RNT in young people with depression and anxiety. The results showed significantly greater reductions in depression, anxiety, and RNT in those who used Mello compared with an inactive control, with moderate to large effect sizes, suggesting that the app may be effective for treating depression and anxiety in youth with elevated RNT. The secondary aim was to explore whether improvements in depression and anxiety were accounted for by reductions in the target transdiagnostic mechanism of RNT, with exploratory analyses supporting this theoretical mechanism of action. Findings concerning feasibility and acceptability were positive. Recruitment targets were exceeded in the allocated trial timeframe, with 86% (55/64) of identified eligible young people participating in the trial and 95% (52/55) of participants completing all assessments. There was high endorsement of the app among users, with 96% (27/28) reporting that they would recommend the app to others and 71% (20/28) rating it as 4 or 5 stars out of 5. Finally, use statistics showed high levels of sustained engagement with Mello over the intervention period, with young people using the app on average half of all days, and 59% (17/29) of users remaining engaged at week 6. These positive results support the promise of Mello as a potentially effective, engaging, and scalable digital intervention.

Systematic reviews have demonstrated generally high levels of acceptability of mental health apps in youth populations across the domains of usability, functionality, and general satisfaction [32,33]. A systematic review of 15 mental health apps for youth [59] revealed an average rating of 3.59 on the Mobile Application Rating Scale [52]. Mello exceeded this rating across all domains, particularly for esthetic appeal, information, functionality, and overall app quality, suggesting that the app was highly acceptable to users. With regard to feasibility, a recent systematic review of RCTs of mental health apps for youth found average dropout rates of approximately 20% for intervention groups [33]. The retention rates in this trial were substantially higher, with only 3% (1 participant) of the Mello group being lost to follow-up. This suggests that the trial was feasible to run and supports the acceptability of the intervention.

While contextualizing rates of engagement with Mello relative to the broader field is complex, given challenges in standard measurement [33,60], the pattern of use within this trial suggests a high level of engagement. Adherence rates of mental health apps within trial contexts have ranged from 65% to 83% [32], with lower estimates among apps used “in the wild” (0.5%-28.6%) [60]. The rate of engagement with Mello, defined as the number of users still active at 3 (26/29, 90%) and 6 (17/29, 59%) weeks after the intervention, as well as the average proportion of days in which Mello was used (20.66/42, 49%), was relatively high compared with previous findings. Consistent with previous research [33], the use of the Mello app decreased over time, and there was no relationship between use and clinical outcomes. This further confirms the typical reduction in engagement over time observed in digital interventions [61], which is also observed in face-to-face services [7], as well as the complexities underpinning the relationship between engagement and clinical outcomes. Although it is important for individuals to use digital interventions to receive benefits, the degree to which long-term engagement is associated with linear clinical improvement over time remains unclear. For example, if a digital intervention is successful, then an individual may come to rely on it less over time, reflecting a decrease in engagement. Further research is needed to unpack the complex relationship between engagement and effectiveness [62].

Although a larger trial is needed to confirm the results, the effect sizes show that using Mello led to reductions in depression and anxiety. Our findings suggest that this may occur via the hypothesized mechanism of reducing RNT. If confirmed, these findings support the theory that targeting reductions in transdiagnostic mechanisms will lead to improvements in symptoms [14]. A recent meta-analysis of psychological treatments for depression, anxiety and RNT in youth populations found moderate between-group effect sizes ranging from 0.42 to 0.47 across these outcomes (Cohen *d*) [22]. The primary effects of Mello compare well with these estimates for all outcomes, particularly for anxiety (Cohen *d*=0.61) and RNT (Cohen *d*=0.87) and to a lesser degree for depression (Cohen *d*=0.50). These effects also compare well with pooled effects from meta-analyses of mental health apps for depression (Hedges *g*=0.34−0.38) [31,63] and anxiety (Hedges *g*=0.33−0.43) [31,64]. Considering that the levels of depression and anxiety experienced by young people in this trial were moderate to severe according to validated measures (PHQ-9 and GAD-7) and that Mello was a self-guided app with only minimal weekly support calls, these effects suggest that the app has the potential to be a scalable, cost-effective intervention. Furthermore, given that 71% (39/55) of the sample was already receiving mental health treatment and this was counterbalanced across groups, these effects could be viewed as being in addition to treatment received by most of the sample. It is important to note that, although the weekly phone calls were not therapeutic in nature, participants may have built rapport with the researcher, resulting in increased engagement. Nonetheless, these results challenge previous findings suggesting that primarily self-guided apps may have limited effectiveness in improving mental health outcomes [31] and highlight the important potential for personalized, transdiagnostic apps to provide accessible, cost-effective, and scalable evidence-based mental health support for young people.

The pattern of effect sizes across outcome measures provides insights into the potential therapeutic mechanisms of Mello. On the basis of a recent meta-analysis [22], app content emphasized “third wave,” process-focused intervention activities (eg, defusion and mindfulness), as these interventions may be more effective at reducing RNT in this population alongside content-focused activities. The effect on RNT observed in this trial (Cohen *d*=0.87) is consistent with the pooled effect of process-focused interventions (Cohen *d*=0.85) found in the meta-analysis, which included treatments such as mindfulness and acceptance and commitment therapy [65]. The effects on secondary outcomes lend support to the theoretical benefit of process-based approaches for reducing RNT in Mello, with cognitive defusion, a core process-focused technique involving observing and conceptualizing negative thoughts in an objective, nonjudgmental way [66], also had a moderate to large effect size (Cohen *d*=0.67). In contrast, cognitive reappraisal, a cognitive technique that targets modifying the content of negative thoughts [67], did not have any significant effects. This may be further supported by the finding of large improvements in participant-reported confidence in managing patterns in negative thinking patterns but no improvement in RNT pattern awareness. Similar mechanisms of change have been demonstrated in other third-wave approaches, such as meta-cognitive therapy, which focuses on adapting beliefs and responses to negative thoughts rather than challenging their validity [68]. A fully powered trial is needed to establish the causal mechanisms of Mello, and a direct comparison between these measures was not explored; however, this emerging picture suggests that the intervention may have exerted its effects primarily by improving the relationship young people have with their negative thoughts rather than by changing thought content. However, although group trends may reveal the most clinically effective approaches for the “average” young person, Mello was developed based on the foundation that greater effects may be observed when interventions are tailored to the individual. Consistent with the view of clinicians on the need for flexibility and individualization when delivering psychological treatments in routine practice [29], offering a suite of intervention options and supporting young people to learn what works for them will continue to inform future research and development for Mello.

A key feature of Mello, consistent with the design of JITAIs [39], is the use of an algorithm to tailor recommendations for therapy activities based on responses to EMA check-ins. This emphasizes the personalized nature of the intervention, which not only targets a mechanism relevant to problems experienced by users but also the relevance of strategies to the context and experiences in the moment. This level of personalization may have contributed to the high levels of engagement with and acceptability of Mello. A primary focus of future research and development for Mello will be to further increase the level of personalization using machine learning. Specifically, a future version of Mello could continually learn what strategy for reducing RNT works best for that individual young person at that specific time and place. This form of dynamic tailoring, whereby the intervention is continually adapted based on assessments over the course of the intervention, has been found to be more effective at improving health behaviors than those based on “static” assessments where the same “rule” is applied in each instance [41,69]. Thus, the role of passive sensors and adaptive tailoring using artificial intelligence and machine learning has been proposed as key to the future of digital interventions, particularly JITAIs [39].

### Limitations

There were several important limitations in this study. First, a fully powered trial is needed to confirm the results due to the small sample size, including the causal pathways of the intervention using formal mediation analyses across multiple time points and subgroup analysis to examine how the effects may differ between groups (eg, those who are and are not receiving mental health treatment). Second, it is unknown whether the effects were maintained because there was no follow-up time point. Third, the primary outcomes were measured using self-report scales during unblinded outcome assessments, raising possible sources of bias. Fourth, Mello users, but not the control group, received brief weekly phone calls, and although these were not therapeutic in content, they could not be ruled out as contributing to the effects. It is highly possible that these calls contributed to engagement, and considering this support may not be scalable, engagement would likely be lower in the “real world” [60]. Furthermore, the “digital placebo effect” [70], whereby the use of a novel digital product may contribute to improvements in symptoms beyond the active ingredients of the treatment, was not controlled for. A trial design with an active control group involving the use of a therapeutically inactive mobile app, as well as supportive phone calls occurring consistently across groups, is therefore needed.

### Conclusions

The results of this pilot RCT suggest that the Mello app is feasible, acceptable, and potentially clinically effective. Large and significant reductions in RNT scores were found, which may explain the effects of Mello on anxiety and depression. These findings support ongoing research and development, with a focus on enhancing personalization using machine learning, conducting a larger RCT to establish efficacy and mechanisms of action, evaluating cost-effectiveness, and ultimately scaling the intervention to ensure that all young people have permanent access to Mello.

## Appendix

Multimedia Appendix 1 Participant information and consent form.

Multimedia Appendix 2 Description of therapy activities.

Multimedia Appendix 3 CONSORT-eHEALTH checklist (V 1.6.1).

Multimedia Appendix 4 Ratings of therapy activity helpfulness.

Multimedia Appendix 5 Digital Working Alliance Inventory results.

Multimedia Appendix 6 Engagement correlations.

Multimedia Appendix 7 Secondary outcome results.

## Abbreviations

CFQ: Cognitive Fusion Questionnaire
CONSORT: Consolidated Standards of Reporting Trials
EMA: ecological momentary assessment
GAD-7: Generalized Anxiety Disorder Scale 7
JITAI: just-in-time adaptive intervention
PHQ-8: Patient Health Questionnaire 8
PTQ: Perseverative Thinking Questionnaire
RCT: randomized controlled trial
REDCap: Research Electronic Data Capture
RNT: repetitive negative thinking
RRS: Ruminative Response Scale
WEMWBS: Warwick-Edinburgh Mental Well-Being Scale

## Data Availability

The data sets generated and analyzed in this study are available from the corresponding author upon reasonable request.
