# Supplementary material for: A Personalized, Transdiagnostic Smartphone Intervention (Mello) Targeting Repetitive Negative Thinking in Young People With Depression and Anxiety: Pilot Randomized Controlled Trial
Source: J Med Internet Res. 2023 Dec 13;25:e47860. doi: 10.2196/47860 (PMC10753417; doi:10.2196/47860)
Supplement: Multimedia Appendix 1 [file jmir_v25i1e47860_app1.pdf]

Mello Study Participant Information and Consent Form (self)

Plain Language Statement

Centre for Youth Mental Health

***Project:* Pilot randomised controlled trial of a personalised, transdiagnostic smartphone intervention targeting repetitive negative thinking to reduce depression and anxiety in young people – young people**

Professor Mario Alvarez-Jimenez (Responsible Researcher)
Tel: +61 3 9966 9383 Email: mario.alvarez@orygen.org.au

Participant ID: ____________________________

Study Title: Pilot randomised controlled trial of a personalised, transdiagnostic smartphone intervention targeting repetitive negative thinking to reduce depression and anxiety in young people - young people

Project ID: 21316

Project Sponsor: Orygen, The National Centre of Excellence in Youth Mental Health

Coordinating Principal Investigator/ Principal Investigator: Professor Mario Alvarez-Jimenez

Associate Investigator(s): Dr Imogen Bell (associate researcher), Tamsyn Gilbertson (associate researcher), Dr Simon D'Alfonso (associate researcher), Jennifer Nicholas (associate researcher), Mx Lee Valentine (associate researcher), Ms Shaunagh O'Sullivan (associate researcher), Dr Nicola Chen (associate researcher), Dr Chelsea Arnold (project manager), Ms Emily Castagnini (research assistant)

Introduction

Thank you for your interest in participating in this research project. The following few pages will provide you with further information about the project, so that you can decide if you would like to take part in this research.

Please take the time to read this information carefully. You may ask questions about anything you don’t understand or want to know more about.

Your participation is voluntary. If you don’t wish to take part, you don’t have to. If you begin participating, you can also stop at any time.

What is this research about?

Many young people struggle with feelings of depression and anxiety One of the possible causes of anxiety and depression is getting caught up in thinking about negative feelings or that things aren’t going right. Getting stuck in these thoughts can be unhelpful and make you feel worse. We may be able to help young people feel less depressed and anxious by giving them skills to get unstuck from negative thoughts. Research has shown that these skills can be effective, but they are often hard to learn and remember to use when stuck thinking is happening. Psychological therapy can also be hard to access and difficult for some young people to engage with.

Smartphone apps might be able to help young people with stuck thinking by giving them support to learn and practice skills to get unstuck from negative thoughts and emotions in daily life when it’s happening. We are conducting this study to evaluate a new smartphone app called ‘Mello’ that is designed to help improve depression and anxiety in young people by helping them get unstuck from negative thoughts using evidence based, guided activities. This research study will evaluate whether Mello is helpful for young people, what their experiences are like with the app, and if it’s feasible to use.

What will I be asked to do?

Firstly, a researcher will ask you some questions over the phone to screen if you are eligible for the study. If you are eligible and agree to participate, we will ask you to attend a 1.5 hour online session over Zoom with the researcher. Note that eligibility will need to be re-confirmed within this session, therefore participation can only be confirmed at this point. If it is determined that you are ineligible at this point, you will still be reimbursed for your time in attending this session.
The session will involve going through informed consent and signing it if you decide to participate, and then completing some questionnaires. These questionnaires will ask some basic information about you, such as your gender, age, history of using mental health services, and recent mental health experiences such as symptoms of depression and anxiety.

All participants will complete two additional assessments lasting approximately 1.5 hours online over Zoom – one at 3 weeks after informed consent and another approximately 6-8 weeks after informed consent. These assessments will involve completing the same questionnaires about recent mental health experiences, and additional questions about experiences with Mello for those who use the app. Participants who use Mello will also be invited to attend an optional one hour, one-on-one interview which will be audio recorded to ask about experiences with Mello after the final assessment session. The total length of participation is between 6-8 weeks.

After the first assessment, you will be randomly allocated to either receive access to the Mello app for 6 weeks or a control group who does not get access to Mello. This is so that we can compare a group of young people who receive the intervention to a group of young people who don't, to see if Mello is more beneficial that receiving no intervention at all. All participants will receive weekly phone calls lasting approximately 5 – 10 minutes to check in on their wellbeing (control group) and support their use of the app (intervention group).

Mello group

If you receive access to Mello, the researcher will make a time to meet with you and download the app onto your phone and show you how to use it. Mello will then be available for you to use for a total of 6 weeks.

The features of Mello include:

1) Monitoring stuck thinking, moods, activities and locations via three check in questions in the app. You will be prompted three times a day to complete a check in or you can choose to complete a check in at any time in the app. Check ins will take approximately less than 1 minute to complete.
2) Tailored therapy activities designed to help get unstuck from negative thoughts in the moment, which are tailored based on your answers to check ins or you can access them at any time in another section of the app. Therapy activities will take approximately 5 minutes to complete.
3) Summary of check in information that shows you the relationship between your stuck thinking and mood, activity and location.

Mello has been designed by experts in digital mental health and treatments for depression and anxiety in young people, with input from young people and clinicians outside the research group. Each activity is guided via audio or text and involves doing things like answering forms in the app to challenge negative thoughts or practice a mindfulness exercise. Each activity has been designed based on research evidence with input from young people and clinicians.

Control group

The control group will not receive access to Mello and will instead only complete the assessment sessions and phone calls with researchers.

All participants will receive weekly phone calls from researchers to check on their experiences with Mello and help trouble shoot any issues, or for a brief wellbeing check for participants in the control group.

Participants are able to continue any additional treatment they may be receiving for the duration of their participation in the research study. Mello will not interfere with this treatment. You may wish to discuss your participation in this research study and inform your treating clinician, if you have one, that you are involved in Mello by showing them this information and consent form.

Note that upon completion of this study, participants will not be able to continue accessing the Mello intervention. This is because the app will require updating and a plan for ongoing maintenance at this point. However we do plan to make Mello available to all young people within the next 2 years.
A research team member or a representative of the Orygen communications team may contact you asking for your thoughts on the research project or your lived experience. You are free to say no to their requests, or to provide comments anonymously. Your comments may be used to promote the research study's findings on social media, in a news article or other communications materials. If you do not wish to be contacted for communications purposes, please let a member of the study team know.

What are the possible benefits?

We cannot guarantee that you will receive any direct benefits from this research, however Mello has been designed to help improve depression and anxiety in young people. This is the first ever study of Mello, so we can't guarantee that if you receive access to Mello, that you will see improvements in these symptoms. You may find benefit from knowing you are contributing to helping to develop new treatments, therefore potentially helping others in future.

Participants will be reimbursed $45 for your time for each assessment session, and an additional $30 for the optional interview if they use Mello. This means the total reimbursement for this research study is $135, plus a potential extra $30.

What are the possible risks?

You may feel that some of the questions you are asked during the research are stressful or upsetting. If you do not wish to answer a question, you may skip it and go to the next question, or you may stop immediately.

Because Mello is an intervention for depression and anxiety, it is possible that working through the activities or answering the check in questions becomes upsetting or distressing. If this occurs, you can choose to stop using Mello at any time. The details listed on the final page of this form also provides the contact details of the researchers involved should you experience any unwanted distress from your involvement in this research, as well as details of emergency services. We will ask you each week during phone calls about whether you are experiencing any unwanted effects from your involvement in the trial, including your use of Mello, and will help you make a decision about whether to continue or if you are in need of support services. We will ask you to provide details of an emergency contact person in the case of any concerns about your wellbeing or that of others.

Do I have to take part?

No. Participation is completely voluntary. You are able to withdraw at any time.
Your decision whether to take part or not to take part, or to take part and then withdraw, will not affect your access to any service you may be receiving care from. Please get in touch with the researchers listed on the final page of this form via phone or email if you wish to withdraw from the study.

Will I hear about the results of this project?

If you'd like, we will email you a short summary of the results once the project has finished. We will also publish results of the research project in scientific journals and on the Orygen website.

What will happen to information about me?

By signing the consent form you consent to the study research staff collecting and using personal information about you for this research project. Any information obtained in connection with this research project that can identify you will be treated as confidential and securely stored. It will be disclosed only with your permission, or as required by law.

Information about you collected for this research will be stored using a unique code on secure electronic servers at Orygen. No personally identifiable information about you (for example, your name or this Consent Form) will kept together in the same file with this de-identified research information. Information collected for this study will only be re-identifiable using a coding system, which will be stored in a separate password-protected computer file at Orygen.

Personally identifying information will be kept for a minimum of 15 years (17 years in the case of young people aged 16 years), but may be kept indefinitely, after the results of this study have been published. Anonymous information provided might be kept indefinitely in a secure computer file at Orygen. We will ask your permission now to use this data in future research studies. By making this data easier to share, researchers hope to learn new and important things more quickly than before.
Any future research projects which seek to use your data must have been reviewed and approved by a Human Research Ethics Committee

Any information obtained during this research project is subject to inspection (for the purpose of verifying the procedures and the data) by the relevant authorities and authorised representatives of Orygen and The University of

Melbourne Human Research Ethics Committee, or as required by law. By signing the Consent Form, you authorise release of, or access to, this confidential information to the relevant study personnel and regulatory authorities as noted above.
It is anticipated that the results of this research project will be published and/or presented in a variety of forums. In any publication and/or presentation, information will be provided in such a way that you cannot be identified, except with your permission.

In accordance with relevant Australian and Victorian privacy and other relevant laws, you have the right to request access to your information collected and stored by the research team. You also have the right to request that any information with which you disagree be corrected. Please contact the research team member named at the beginning of this document if you would like to access your information.

If any member of the research team becomes aware of any signs that you may be at risk of harm to yourself or someone else, a number of steps may be taken by members of the research team to ensure your safety. These may include contacting emergency or crisis services, and/or contacting your emergency contact. You will be kept informed of any steps taken in response to indications of risk.

Who is funding this project?

Funding for this project is provided by the Telstra Foundation Tech4Good Challenge.

Where can I get further information?

If you would like more information about the project or are in need of immediate support, please contact the researcher: Professor Mario Alvarez-Jimenez (+61 9966 9383)

If you are experiencing distress and require emergency support, please contact Lifeline on 13 11 14.

Who can I contact if I have any concerns about the project?

This project has human research ethics approval from The University of Melbourne (Project ID: 21316). If you have any concerns or complaints about the conduct of this research project, which you do not wish to discuss with the research team, you should contact the Research Integrity Administrator, Office of Research Ethics and Integrity, University of Melbourne, VIC 3010. Tel: +61 8344 1376 or Email: research-integrity@unimelb.edu.au. All complaints will be treated confidentially. In any correspondence, please provide the name of the research team and/or the name or ethics ID number of the research project.

Emergency contacts

If you require emergency assistance please contact one of the following services (available 24/7):

• Lifeline on 13 11 14
• Suicide line on 1300 651 251
• The Youth Access Team on 1800 888 320

Consent Form

Centre for Youth Mental Health

THE UNIVERSITY OF MELBOURNE

***Project:* Pilot randomised controlled trial of a personalised, transdiagnostic smartphone intervention targeting repetitive negative thinking to reduce depression and anxiety in young people – young people**

Please check all the boxes below to indicate that you consent and understand the following:

	I agree
1. I consent to participate in this project, the details of which have been explained to me, and I have been provided with a written plain language statement to keep.	○
2. I understand that the purpose of this research is to evaluate a new smartphone intervention	○
3. I understand that my participation in this project is for research purposes only.	○
4. I acknowledge that the possible effects of participating in this research project have been explained to my satisfaction.	○
5. In this project I will be required to attend three, 1.5 hour assessment sessions and potentially receive access to a smartphone intervention for 6 weeks or a control group who will not receive access to the smartphone intervention	○
6. I understand that any interview I am involved in may be audio recorded	○
7. I understand that my participation is voluntary and that I am free to withdraw from this project anytime without explanation or prejudice and to withdraw any unprocessed data that I have provided.	○
8. I understand that the data from this research will be stored at the University of Melbourne and will be destroyed after 15 years (17 years in the case of young people aged 16 years), unless I consent to indefinite storage	○

9. I understand that my data may be used for closely related research projects in future	◯	
10. I have been informed that the confidentiality of the information I provide will be safeguarded subject to any legal requirements; my data will be password protected and accessible only by the named researchers.	◯	
11. I understand that after I sign and return this consent form, it will be retained by the researcher.	◯	

Please select 'yes' or 'no' to indicate whether or not you agree to participate in the following additional optional components of the research:

	Yes	No
I agree to being contacted to potentially take part in an interview about Mello (optional)	◯	◯
I consent to my data being stored indefinitely to use in future research at Orygen (optional)	◯	◯
I consent to researchers contacting me about future research opportunities. I recognise that I am not obliged to participate should I be contacted and that I can request for my details to be removed at any time (optional).	◯	◯
I consent to researchers or members of the media team to contact me about my experiences of the research project. I recognise that I am not obliged to share these experiences should I be contacted and that I can request for my details to be removed at any time (optional).	◯	◯

I would like to be emailed a summary of the results of the project once it has finished (optional).	○	○

Participant name: ______________________________

Please sign here to confirm you consent by clicking 'add signature' on the right here -->

You can draw your signature using your mouse/cursor or your finger if using a touchscreen, then press 'save signature' ______________________________________

Date signed: ______________________________
