# Supplementary material for: A Personalized, Transdiagnostic Smartphone Intervention (Mello) Targeting Repetitive Negative Thinking in Young People With Depression and Anxiety: Pilot Randomized Controlled Trial
Source: J Med Internet Res. 2023 Dec 13;25:e47860. doi: 10.2196/47860 (PMC10753417; doi:10.2196/47860)
Supplement: Multimedia Appendix 2 [file jmir_v25i1e47860_app2.docx]

**Description of therapy activities in Mello**

| **Therapy Activity** | **Description** |
| --- | --- |
| Slowing Down  (6-8 mins) | **Slow Breathing:** Basic breathing de-arousal techniques involving 4-2-6 slow breathing |
| Noticing Thoughts  (3-5 mins) | **Defusion – visual: Mindful visualisation exercise to let go of unhelpful thoughts and let them pass naturally** |
| Airing Out  (2-3 mins) | Emotional Expression: Unstructured space to express difficult emotions and thoughts in concrete form. |
| Connecting to Senses (3-5 mins) | **Grounding 5 Senses:** Short mindfulness skill attending to external sensory info to anchor thoughts in the present. |
| Reaching Out  (5-6 mins) | **Social Support:** Generating potential supports, and drafting messages to access them. |
| Thinking Time  (4-6 mins) | **Worry Time:** Two-part behavioural experiment, a) postponing worry, and b) using CBT-prompts to challenge thoughts at designated time. |
| Changing Perspectives (10-12 mins) | **Bias Modification:** Guided CBT-style prompts to challenge either depressive-type rumination or anxiety-type worry. |
| Solving Puzzles  (10-12 mins) | **Problem Solving: Structured exercise defining problem, generating solutions and planning actions.** |
| Taking Stock  (3-4 mins) | Gratitude: **Reflecting on positive factors and things one is grateful for.** |
| Being Kind  (5-6 mins) | **Self-Compassion:** Responding to inner critic with compassion, and generating alternate thoughts based on what you would tell a friend. |
| Reflecting on Positives (5-6 mins) | **Cultivating Positive Emotions:** Recalling recent positive events, interactions and emotions when mood is negative. |
| Looking Back  (5-6 mins) | Emotional awareness: Reflection activity to promote emotional awareness through highlighting changing perspectives over time |
