# Supplementary material for: A Personalized, Transdiagnostic Smartphone Intervention (Mello) Targeting Repetitive Negative Thinking in Young People With Depression and Anxiety: Pilot Randomized Controlled Trial
Source: J Med Internet Res. 2023 Dec 13;25:e47860. doi: 10.2196/47860 (PMC10753417; doi:10.2196/47860)
Supplement: Multimedia Appendix 4 [file jmir_v25i1e47860_app4.docx]

**Ratings of helpfulness by Therapy Activity (range 0-3)**

| Therapy activity | n | M (SD) |
| --- | --- | --- |
| Airing Out | 49 | 1.88 (0.81) |
| Being Kind | 27 | 2.07 (0.47) |
| Changing Perspective | 15 | 2.13 (0.52) |
| Connecting to Senses | 16 | 2.31 (0.70) |
| Looking Back | 20 | 1.75 (0.91) |
| Noticing your Thoughts | 34 | 1.74 (0.79) |
| Reaching Out | 15 | 1.73 (0.70) |
| Reflecting on Positives | 36 | 1.81 (0.71) |
| Slowing Down | 37 | 2.05 (0.47) |
| Solving Puzzles | 13 | 2.00 (0.71) |
| Taking Stock | 77 | 1.81 (0.69) |
| Thinking Time | 23 | 1.78 (0.60) |
| All Activities | 362 | 1.89 (0.70) |

0 = not helpful, 1 = a little helpful, 2 = moderately helpful, 3 = very helpful
