# Supplementary material for: A Personalized, Transdiagnostic Smartphone Intervention (Mello) Targeting Repetitive Negative Thinking in Young People With Depression and Anxiety: Pilot Randomized Controlled Trial
Source: J Med Internet Res. 2023 Dec 13;25:e47860. doi: 10.2196/47860 (PMC10753417; doi:10.2196/47860)
Supplement: Multimedia Appendix 5 [file jmir_v25i1e47860_app5.docx]

**Digital Working Alliance Inventory (D-WAI) item scores (range 1 – 7)**

| **Item description (n = 28)** | **M (SD)** |
| --- | --- |
| I trust the app to guide me towards my personal goals  I believe the app tasks will help me to address my problem  The app encourages me to accomplish tasks and make progress  I agree that the tasks within the app are important for my goals  The app is easy to use and operate  The app supports me to overcome challenges | 4.5 (1.2)  4.8 (1.4)  4.7 (1.6)  4.8 (1.5)  6.1 (1.2)  4.7 (1.4) |
