# Supplementary material for: A Personalized, Transdiagnostic Smartphone Intervention (Mello) Targeting Repetitive Negative Thinking in Young People With Depression and Anxiety: Pilot Randomized Controlled Trial
Source: J Med Internet Res. 2023 Dec 13;25:e47860. doi: 10.2196/47860 (PMC10753417; doi:10.2196/47860)
Supplement: Multimedia Appendix 6 [file jmir_v25i1e47860_app6.docx]

**Correlations between clinical change scores and engagement indices**

| **Engagement index (n = 29)** | **PTQ** | | **PHQ-8** | | **GAD-7** | |
| --- | --- | --- | --- | --- | --- | --- |
|  | ***r*** | ***P*** | ***r*** | ***P*** | ***r*** | ***P*** |
| Therapy activities launched  Check-ins completed  Distinct days of app use  Proportion of days using app | -.26  .27  .26  .26 | .186  .156  .170  .171 | -.17  .29  .23  .23 | .388  .135  .224  .225 | -.14  .33  .36  .36 | .473  .078  .058  .058 |

*PTQ* Perseverative Thinking Questionnaire, *PHQ-8* Patient Health Questionnaire 8, *GAD-7* Generalised Anxiety Disorder 7.
