# Supplementary material for: A Personalized, Transdiagnostic Smartphone Intervention (Mello) Targeting Repetitive Negative Thinking in Young People With Depression and Anxiety: Pilot Randomized Controlled Trial
Source: J Med Internet Res. 2023 Dec 13;25:e47860. doi: 10.2196/47860 (PMC10753417; doi:10.2196/47860)
Supplement: Multimedia Appendix 7 [file jmir_v25i1e47860_app7.docx]

**Graphical results for secondary outcomes per condition over time**


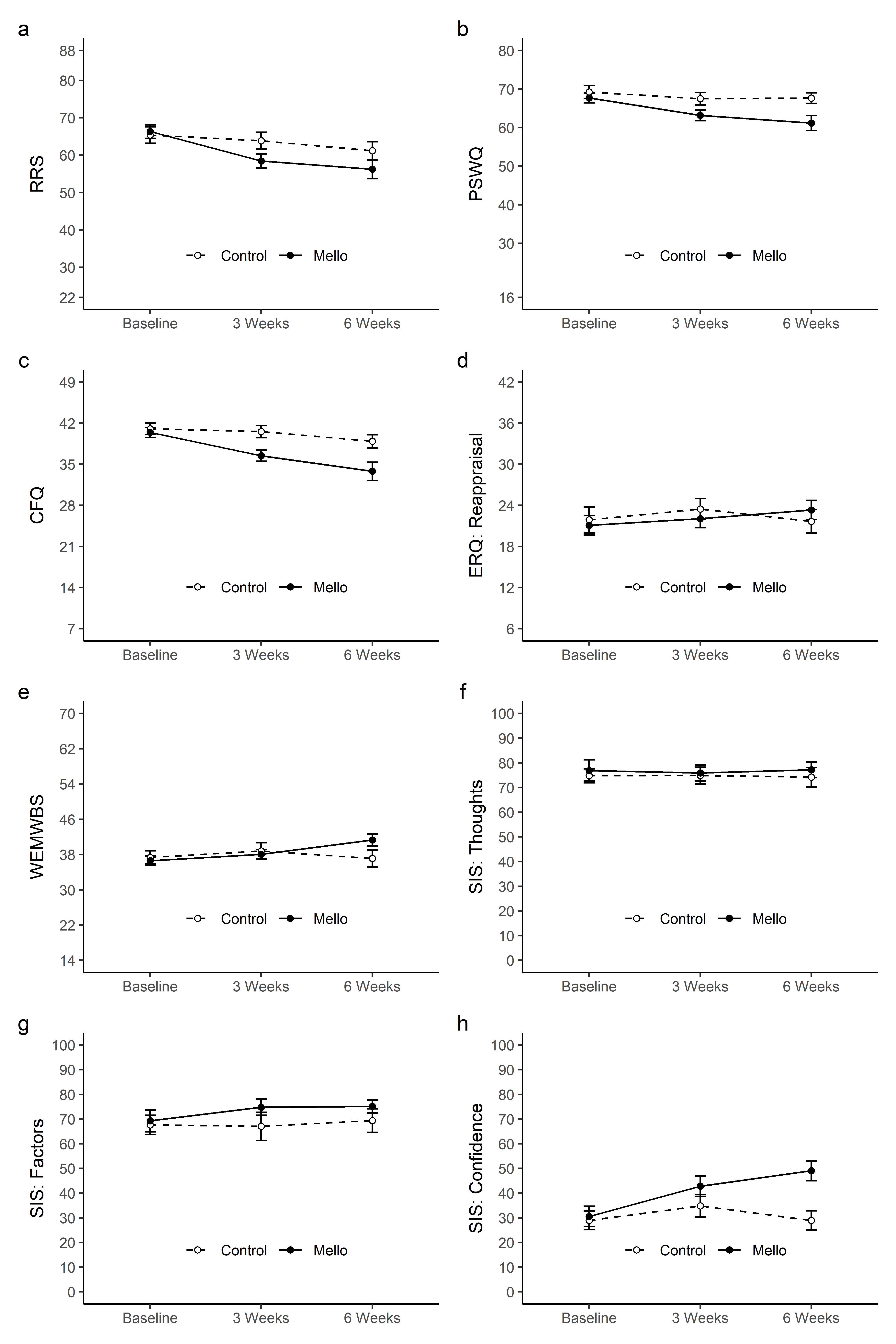


*RRS* Ruminative Response Scale, *PSWQ* Penn State Worry Questionnaire, *CFQ* Cognitive Fusion Questionnaire, *ERQ reappraisal* Emotion Regulation Questionnaire subscale, *WEMWBS* Warwick-Edinburgh Mental Well-being Scale, *SIS* Single item self-awareness and self-management measures.
